# Supplementary material for: Strengthening provider accountability: A scoping review of accountability/monitoring frameworks for quality of RMNCH care
Source: PLOS Glob Public Health. 2023 Nov 9;3(11):e0001968. doi: 10.1371/journal.pgph.0001968 (PMC10635430; doi:10.1371/journal.pgph.0001968)
Supplement: S1 Appendix — (DOCX) [file pgph.0001968.s001.docx]

**S1 Appendix. Global monitoring initiatives included in the review**

(1) WHO’s quality, equity and dignity (QED) network: This initiative focused specifically on monitoring QoC of RMNCH services. Many countries also used its accountability/monitoring framework to develop their own QI frameworks.

(2) Countdown to 2030: This framework was a follow-up to the Countdown to 2015, which tracked the progress of life-saving interventions for reproductive, maternal, newborn, child and adolescent health and nutrition (RMNCAH+N). It focuses on strengthening country evidence and analytical capacity.

(3) The Global Strategy for Women, Children’s, and Adolescents’ health (Every Woman Every Child [EWEC] Network): As stated by the United Nation Children Fund (UNICEF), the Global Strategy (2016-2030) is “a roadmap to achieve right to the highest attainable standard of health for all women, children and adolescents –to transform the future and ensure every newborn, mother and child not only survives, but thrives.” The launch of EWEC aimed to facilitate the attainment of sustainable development goals (SDGs) related to maternal and child health in 2030.

(4) Global Reference List of 100 core health indicators: This list of the indicators is mainly used to monitor the progress towards SDGs. Not all the indicators in the list are for RMNCH. In this study, we extracted any indicators related to RMNCH services from the list.
