# Supplementary material for: Strengthening provider accountability: A scoping review of accountability/monitoring frameworks for quality of RMNCH care
Source: PLOS Glob Public Health. 2023 Nov 9;3(11):e0001968. doi: 10.1371/journal.pgph.0001968 (PMC10635430; doi:10.1371/journal.pgph.0001968)
Supplement: S2 Appendix — (DOCX) [file pgph.0001968.s002.docx]

**S2 Appendix. Definition of the six dimensions of quality of care**

1. Safe – delivering health care that minimizes risks and harm to service users, including avoiding preventable injuries and reducing medical errors;
2. Effective – providing services based on scientific knowledge and evidence-based guidelines;
3. Timely – reducing delays in providing and receiving health care;
4. Efficient – delivering health care in a manner that maximizes resource use and avoids waste;
5. Equitable – delivering health care that does not differ in quality according to personal characteristics such as gender, race, ethnicity, geographical location or socioeconomic status;
6. People-centered – providing care that considers the preferences and aspirations of individual service users and the culture of their community.
