# Supplementary material for: Strengthening provider accountability: A scoping review of accountability/monitoring frameworks for quality of RMNCH care
Source: PLOS Glob Public Health. 2023 Nov 9;3(11):e0001968. doi: 10.1371/journal.pgph.0001968 (PMC10635430; doi:10.1371/journal.pgph.0001968)
Supplement: S3 Appendix — (DOCX) [file pgph.0001968.s003.docx]

**S3 Appendix**

*Table A. Eight quality components in QED framework*

| **1.Evidence based practices** | Decision to refer made without delay |
| --- | --- |
| Women outcome measures | Referral follows predetermined plan without delay |
| Women receive routine assessment and appropriate care | Appropriate information exchange between facilities |
| Women with pre-eclampsia/ eclampsia | **4. Effective communication** |
| Women with postpartum haemorrhage | Women and families receive information about care and have effective interactions with staff |
| Women with delayed or obstructed labour | Coordinated care, with clear, accurate information exchange |
| Women in preterm labour | **5.Respect and preservation of dignity** |
| Women with or at risk for infections | Privacy around the time of labour and childbirth, and their confidentiality is respected |
| Women and newborns: prevent hospital-acquired infections | Not subjected to mistreatment |
| Women (and newborns): harmful practices | Informed choices about the services |
| Newborn outcome measures | **6.Emotional support** |
| Newborns receive routine care immediately after birth | Offered option of companion of choice |
| newborns receive routine postnatal care | Support to strengthen her capabilities |
| Newborns who are not breathing spontaneously | **7.Competent, motivated human resources** |
| Preterm and small babies receive appropriate care | Access at all times to skilled birth attendant |
| Newborns with suspected/risk factors for infection | Skilled birth attendants have competence and skills |
| Newborns: harmful practices | Leadership in continuous QI |
| **2. Actionable information systems** | **8.Essential physical resources available** |
| Complete, accurate, standardized medical record | WASH functioning, reliable, safe and sufficient |
| Mechanism for data collection, analysis and feedback | Labour, childbirth and postnatal care appropriately organized |
| **3.Functional referral systems** | Adequate stock of medicines, supplies and equipment |

*Table B. Detailed quality indicators for QED framework*

| **Indicator** | **Type of service^a^** | **Quality type^b^** | **Quality domain^c^** | **Monitoring level^d^** | **Data collection approach^e^** |
| --- | --- | --- | --- | --- | --- |
| Number of maternal deaths (per 100 000 live births in health facility)** | 3 | 3 | 2 | 1 | 1 |
| Number of maternal deaths classified by cause (ICD-MM)** | 3 | 3 | 2 | 1 | 1 |
| % women with specific obstetric complication (PPH, PE/E, prolonged labour, infection/sepsis) | 3 | 3 | 2 | 1 | 1 |
| Obstetric case fatality rate (disaggregated by direct and indirect causes when possible)** | 3 | 3 | 2 | 1 | 1 |
| Maternal cause-specific case fatality rate (PPH, PE/E, infection/sepsis, prolonged labour) | 3 | 3 | 2 | 1 | 1 |
| % facilities with basic essential equipment and supplies available | 3 | 1 | 2 | 2 | 1 |
| % facilities with written, up-to-date clinical protocols | 3 | 1 | 4 | 2 | 3 |
| % staff with recent in-service training | 3 | 2 | 2 | 1 | 5 |
| % facilities with recent supportive supervision | 3 | 2 | 2 | 2 | 5 |
| % women assessed appropriately at admission in labour [prenatal history/risk factors, vital signs, danger signs, physical examination] | 3 | 2 | 2 | 1 | 3 |
| % women monitored appropriately during labour [*see forthcoming 2018 WHO intrapartum care recommendations*] | 3 | 2 | 4 | 1 | 3 |
| % women with blood pressure, pulse and temperature monitored appropriately [admission, labour, postpartum period] | 3 | 2 | 4 | 1 | 1 |
| % women with appropriate monitoring during postpartum period for danger signs, including bleeding [per local protocol and national/global guidelines] | 3 | 2 | 4 | 1 | 2 |
| % facilities with magnesium sulfate and antihypertensives available | 3 | 1 | 2 | 2 | 5 |
| % women with severe PE/E treated with magnesium sulfate | 3 | 2 | 2 | 1 | 1 |
| % women with PE/E managed appropriately based on maternal/fetal status and gestational age (composite indicator) (see WHO MCPC 2nd edition, 2017 | 3 | 2 | 4 | 1 | 1 |
| % women with pre-eclampsia who progressed to eclampsia PE/E case fatality rate (valid only in high-volume facilities or when aggregated across multiple facilities) | 3 | 2 | 4 | 1 | 1 |
| % facilities with uterotonic drugs available | 3 | 1 | 2 | 2 | 5 |
| % CEmONC facilities with functional blood transfusion service | 3 | 1 | 2 | 2 | 1 |
| % women administered immediate postpartum uterotonic (PPH prevention)* | 3 | 2 | 2 | 1 | 1 |
| % women who developed PPH receiving appropriate treatment (composite indicator, e.g. uteronic, tranexamic acid, uterine balloon tamponade, etc.) (see WHO MCPC 2nd edition 2017) | 3 | 2 | 4 | 1 | 1 |
| PE/E case fatality rate (valid only in high-volume facilities or when aggregated across multiple facilities) | 3 | 3 | 4 | 1 | 1 |
| % facilities with supplies/equipment for vacuum or forceps-assisted delivery | 3 | 1 | 2 | 2 | 5 |
| % women with prolonged labour (active labour > 12 hours) managed appropriately (composite indicator) (see WHO 2018 intrapartum care recommendations) | 3 | 2 | 4 | 1 | 3 |
| % women with prolonged/obstructed labour who gave birth by C-section | 3 | 2 | 2 | 1 | 1 |
| % all women who gave birth in the facility whose active first stage of labour > 12 hours | 3 | 3 | 2 | 1 | 1 |
| % women with obstructed labour with unmet need for C-section | 3 | 3 | 2 | 1 | 1 |
| Case fatality rate for women with prolonged labour (valid only in high-volume facilities or when aggregated across multiple facilities) | 3 | 3 | 2 | 1 | 5 |
| Newborn asphyxia rate (adverse intrapartum outcome) | 3 | 3 | 2 | 1 | 5 |
| % facilities with antenatal corticosteroids available | 3 | 1 | 2 | 2 | 5 |
| % women with preterm pre-labour rupture of membranes who received prophylactic antibiotics | 3 | 2 | 2 | 1 | 2 |
| % preterm newborns whose mothers received corticosteroids when indicated | 3 | 2 | 2 | 1 | 1 |
| % facilities with first- and second-line antibiotics available | 3 | 1 | 2 | 2 | 5 |
| % women with C-section who received prophylactic antibiotics before C-section | 3 | 2 | 2 | 1 | 1 |
| % women with pre-labour rupture of membranes who received antibiotics | 3 | 2 | 2 | 1 | 1 |
| % women who gave birth in the facility with signs of infection treated with appropriate antibiotics | 3 | 2 | 2 | 1 | 1 |
| Maternal infection/sepsis case fatality rate (valid only in large facilities or when aggregated across multiple facilities) | 3 | 3 | 2 | 1 | 1 |
| % uncomplicated, vaginal births where episiotomy performed | 3 | 2 | 2 | 1 | 1 |
| Pre-discharge neonatal mortality rate** | 4 | 3 | 2 | 1 | 1 |
| Facility stillbirth rate (disaggregated by fresh and macerated)** | 4 | 3 | 2 | 1 | 1 |
| Neonatal deaths classified by cause (ICD-PM)** | 4 | 3 | 2 | 1 | 5 |
| Facility intrapartum stillbirth rate (plus fetal heart rate documented at admission) | 4 | 3 | 2 | 1 | 1 |
| % newborns with specific complications (prematurity, possible serious bacterial infection, asphyxia) | 4 | 3 | 4 | 1 | 1 |
| Neonatal cause-specific case fatality rate | 4 | 3 | 4 | 1 | 1 |
| % facilities with essential supplies available | 4 | 1 | 2 | 2 | 5 |
| % facilities with written, up-to-date clinical protocols | 4 | 1 | 4 | 2 | 3 |
| % staff with recent in-service training | 4 | 1 | 2 | 1 | 5 |
| % facilities with supportive supervision | 4 | 2 | 4 | 2 | 1 |
| % newborns breastfed within one hour of birth** | 4 | 2 | 2 | 1 | 5 |
| % newborns with documented birthweight** | 4 | 2 | 2 | 1 | 1 |
| % newborns who received essential early newborn care (drying, skin to skin, delayed cord clamping, breastfeeding) | 4 | 2 | 2 | 1 | 3 |
| % postnatal mothers/babies monitored appropriately for danger signs (vital signs/clinical signs) | 3 | 2 | 4 | 1 | 1 |
| % newborns receiving vitamin K and full vaccination | 4 | 2 | 2 | 1 | 1 |
| % newborns breastfed exclusively at time of discharge | 4 | 2 | 2 | 1 | 1 |
| % postpartum women counselled on birth spacing and postpartum contraception options | 3 | 2 | 4 | 1 | 1 |
| % women discharged postpartum with contraceptive method of choice | 3 | 2 | 4 | 1 | 1 |
| % live births delivered in the facility that were notified by the facility to the civil registrar (in the context where health workers/health facilities have responsibility to notify live birth to the civil registrar) | 3 | 2 | 4 | 1 | 5 |
| % women/families who received postpartum counselling on importance of birth registration and obtaining a birth certificate and the process for registration of their infants with the civil registrar to obtain a birth certificate (applicable for all facilities, regardless of civil registration laws and policies in the country) | 3 | 2 | \| 22 \| \| --- \|   4 | 1 | 1 |
| % live births delivered in the facility that were registered in the civil registry by the facility (applicable where health workers/health facilities have responsibility to register live births into the civil registry) | 3 | 2 | 4 | 1 | 5 |
| % facilities with suction device, mask and bag (size 0 and 1) | 4 | 1 | 4 | 2 | 5 |
| % live-born newborns not breathing after additional stimulation who were resuscitated with bag and mask | 4 | 2 | 2 | 1 | 1 |
| % facilities with supplies/equipment for thermal care and feeding of small babies | 4 | 1 | 2 | 2 | 5 |
| Proportion of newborns < 2000 grams initiated on KMC (or admitted to KMC unit if separate unit exists)** | 4 | 2 | 4 | 1 | 1 |
| % eligible neonatal babies (≤ 2000 grams) who receive near continuous KMC | 4 | 2 | 4 | 1 | 1 |
| % facilities with first- and second-line antibiotics available | 4 | 1 | 2 | 2 | 5 |
| % newborns of mothers with signs of infection who are evaluated for infection and treated as appropriate | 4 | 2 | 2 | 1 | 1 |
| % newborns with signs of infection who received appropriate antibiotics | 4 | 2 | 2 | 1 | 1 |
| % facilities with no displays of infant formula, bottles, teats | 4 | 1 | 5 | 2 | 2 |
| % women who received augmentation of labour (uterotonics) with no indication of delay in labour progress | 3 | 2 | 5 | 1 | 1 |
| % women with uncomplicated, spontaneous vaginal birth in whom episiotomy performed | 3 | 2 | 4 | 1 | 1 |
| % facilities with birth and death registration linked to vital national registration system | 1 | 2 | 7 | 2 | 1 |
| % facilities with standardized registers, patient charts and data collection forms | 1 | 1 | 7 | 2 | 1 |
| % facilities with system for classifying maternal and newborn diseases and health outcomes, including death, aligned with ICD (e.g. ICD-MM/ICD-PM) | 1 | 1 | 7 | 2 | 3 |
| % newborns discharged with accurately completed record | 1 | 2 | 7 | 1 | 1 |
| % newborns with patient identifier and individual clinical medical record | 1 | 2 | 7 | 1 | 1 |
| % postpartum women discharged with accurately completed record | 1 | 2 | 7 | 1 | 1 |
| % facilities in which QI team regularly extracts data, calculates and visualizes prioritized quality indicators | 1 | 2 | 7 | 2 | 5 |
| % facilities where data regularly reviewed and used to make decisions on QI | 1 | 3 | 7 | 2 | 3 |
| % facilities conducted at least one recent review of maternal and perinatal death | 1 | 2 | 7 | 2 | 5 |
| % facilities with standard operating procedures for checking, validating and reporting data | 1 | 1 | 4 | 2 | 3 |
| % maternal deaths reviewed with standard audit tools | 1 | 2 | 7 | 1 | 5 |
| % perinatal deaths reviewed with standard audit tools | 1 | 2 | 7 | 1 | 5 |
| % QED facilities implementing “full” cycle of MPDSR according to WHO technical guidance (maternal and perinatal) (Global MPDSR TWG to consider facility MPDSR assessment tools) | 1 | 2 | 7 | 2 | 5 |
| % facilities with standardized referral protocol for identification, management and referral of women/newborns with complications | 1 | 1 | 5 | 2 | 3 |
| % facilities with supplies for stabilization and pre- referral treatment | 1 | 1 | 5 | 2 | 5 |
| % women/newborns who fulfilled criteria for referral and were referred | 3 | 2 | 5 | 1 | 1 |
| % women/newborns with complications transferred to appropriate care level with referral note | 3 | 2 | 5 | 1 | 5 |
| % women presenting to labour ward who report receiving immediate attention upon arrival | 3 | 2 | 5 | 1 | 2 |
| % facilities with ready access to functioning ambulance or emergency transport | 1 | 1 | 5 | 2 | 5 |
| % facilities with up-to-date list of network facilities providing referral services | 1 | 1 | 5 | 2 | 3 |
| % newborns who died before or during transfer to higher-level facility | 4 | 3 | 5 | 1 | 1 |
| % newborns referred from facility who completed referral | 4 | 3 | 5 | 1 | 1 |
| % pregnant or postpartum women who died before or during transfer to higher-level facility | 3 | 3 | 5 | 1 | 1 |
| % women referred from facility who completed referral | 3 | 3 | 5 | 1 | 1 |
| % facilities with reliable communication methods for referrals and consultation | 1 | 1 | 5 | 2 | 3 |
| % facilities with standardized referral form | 1 | 1 | 5 | 2 | 3 |
| % referred newborns with counter-referral feedback information | 4 | 2 | 7 | 1 | 5 |
| % referred women with counter-referral feedback information | 3 | 2 | 7 | 1 | 5 |
| % facilities with accessible health education materials | 1 | 1 | 6 | 2 | 3 |
| % facilities with written policy to promote interpersonal communication and counselling | 1 | 1 | 6 | 2 | 3 |
| % staff with recent training on interpersonal communication | 1 | 2 | 2 | 1 | 5 |
| % facilities receiving supportive supervision that addresses counselling | 1 | 2 | 4 | 2 | 5 |
| % women receiving postnatal information and counselling before discharge** | 3 | 2 | 4 | 1 | 5 |
| % women who felt they were adequately informed by the health workers about their care, including examinations | 3 | 3 | 4 | 1 | 2 |
| % women who reported they were given an opportunity to discuss their concerns and preferences | 3 | 3 | 6 | 1 | 2 |
| % facilities with standard form for documenting clinical progress and care | 1 | 1 | 4 | 2 | 3 |
| % facilities with written protocols for verbal and written handovers (shift change, intra-facility transfer, referral, discharge) | 1 | 1 | 4 | 2 | 3 |
| % women for whom a partograph has been completed | 3 | 2 | 6 | 1 | 1 |
| % facilities where physical environment allows privacy | 1 | 1 | 6 | 2 | 3 |
| % facilities with written, up-to-date protocols to ensure privacy and confidentiality | 1 | 1 | 6 | 2 | 3 |
| % women reported receiving dignified and respectful care during maternity visit | 3 | 3 | 6 | 1 | 1 |
| % facilities with written accountability mechanism in the event of mistreatment | 1 | 1 | 6 | 2 | 3 |
| % facilities with written, up-to-date zero-tolerance nondiscriminatory policies on mistreatment | 1 | 1 | 6 | 2 | 3 |
| % staff with recent training on respectful care | 1 | 2 | 6 | 1 | 4 |
| % women who gave birth in facility who reported physical or verbal abuse to themselves [or their newborns]** | 3 | 3 | 6 | 1 | 2 |
| % facilities with written, up-to-date policies on obtaining informed consent | 1 | 1 | 6 | 2 | 3 |
| % facilities with standard informed consent form | 1 | 1 | 6 | 2 | 3 |
| % women who felt adequately informed by health workers about their health and care | 3 | 3 | 6 | 1 | 2 |
| % facilities with written, up-to-date policies for one person of woman’s choice | 1 | 1 | 6 | 2 | 3 |
| % facilities with labour and childbirth areas organized to allow for private space | 3 | 1 | 6 | 2 | 5 |
| % women who wanted and had a companion of their choice in labour [childbirth]** | 3 | 2 | 6 | 1 | 2 |
| % women reported receiving supportive care during maternity stay | 3 | 2 | 6 | 1 | 2 |
| % facilities with written, up-to-date protocol on minimizing unnecessary interventions | 1 | 1 | 6 | 2 | 3 |
| % staff with recent training on providing emotional support | 1 | 2 | 6 | 1 | 5 |
| % women undergoing bereavement or adverse outcome who report additional emotional support from facility staff | 3 | 3 | 6 | 1 | 2 |
| % facilities displaying roster of staff on duty, shift times | 1 | 1 | 7 | 2 | 3 |
| % facilities with skilled birth attendant available all the time in sufficient numbers to meet workload | 1 | 1 | 7 | 2 | 5 |
| % available posts that are filled by staff with necessary competence | 1 | 1 | 7 | 2 | 4 |
| % births attended by a skilled birth attendant | 3 | 2 | 2 | 1 | 5 |
| % women reporting sufficient staff at health facility | 3 | 3 | 2 | 1 | 2 |
| % facilities with standard procedures for recruitment, motivation and retention | 1 | 1 | 7 | 2 | 3 |
| % facilities with programme for continuing professional and skills development | 1 | 1 | 7 | 2 | 3 |
| % skilled birth attendant staff with recent in-service training | 3 | 1 | 2 | 1 | 5 |
| % staff who supervised/mentored to support clinical competence and QI in last quarter | 1 | 2 | 2 | 1 | 5 |
| % staff who can identity and report on at least one clinical activity in which they are personally involved | 1 | 2 | 2 | 1 | 5 |
| Measure of health worker experience of providing care in the facility and/or support – *to be determined* | 1 | 2 | 2 | 1 | 4 |
| % facilities with written, up-to-date plan for improving quality of care and patient safety | 1 | 1 | 2 | 2 | 5 |
| % facilities with designated QI team | 1 | 1 | 2 | 2 | 5 |
| % facilities with QI review meeting within at least past one month | 1 | 2 | 2 | 2 | 5 |
| % leaders at facility trained in QI and leading change | 1 | 2 | 2 | 1 | 5 |
| % facilities with mechanism for regular collection of information on patient and provider experiences | 1 | 1 | 7 | 2 | 4 |
| % facilities with an established liaison mechanism to district (and/or national level) on quality issues | 1 | 1 | 7 | 2 | 5 |
| % QI meetings held in last 12 months | 1 | 2 | 7 | 1 | 4 |
| % facilities that participated in data sharing with district and community to inform user decision- making, prioritization and planning | 1 | 2 | 7 | 2 | 5 |
| % leaders communicated performance through established mechanisms (e.g. dashboards) | 1 | 2 | 7 | 1 | 4 |
| % facilities with basic water supply in maternity care areas (labour, birth, postnatal) | 1 | 1 | 4 | 2 | 5 |
| % facilities with basic environmental cleaning practices in maternity areas (labour, birth, postnatal); written cleaning protocols, trained cleaning staff and providers | 1 | 1 | 4 | 2 | 5 |
| % facilities with basic health-care waste management in maternity care areas | 1 | 1 | 4 | 2 | 4 |
| % facilities with basic hygiene provisions in maternity care areas (functional handwashing station, access to bathing/shower area, basic sterile equipment) | 1 | 1 | 4 | 2 | 5 |
| % facilities with basic sanitation available for women during and after labour and childbirth (toilet, latrine) | 1 | 1 | 4 | 2 | 4 |
| % facilities with written protocol and awareness materials (posters) on WASH and waste management | 1 | 1 | 4 | 2 | 4 |
| % women reporting satisfactory access to water | 1 | 3 | 2 | 1 | 2 |
| % facilities with adequate labour and childbirth areas/rooms for estimated number of births | 3 | 1 | 4 | 2 | 3 |
| % facilities with dedicated area in labour/childbirth area for resuscitation of newborns, which is adequately equipped | 3 | 1 | 4 | 2 | 3 |
| % facilities with policy and space for rooming-in of mothers and babies 24 hours a day | 3 | 1 | 4 | 2 | 3 |
| % women reporting clean physical environment | 3 | 3 | 2 | 1 | 2 |
| % facilities with regular source of electricity | 1 | 1 | 4 | 2 | 2 |
| % facilities with essential laboratory supplies and tests | 1 | 1 | 2 | 2 | 3 |

a: 1= General; 2 = Reproductive; 3 = Maternal; 4 = newborn; 5 = Child

b: 1= Inputs; 2 = Outputs; 3 = Outcomes

c: 1= Efficiency; 2 = Effectiveness; 3 = Equity, 4 = safety; 5 = timeliness; 6 = Patient centered; 7 = Cannot be categorized

d: 1 = Facility level; 2 = district/reginal level

e: 1 = Routine information system; 2 = Client interview; 3 = Observation; 4 = others; 5 = multiple approach

*Table C. Monitoring indicators from Countdown to 2030, Core 100 health indicators, and Global Strategy*

| **Indicator** | **Type of service^a^** | | **Quality type^b^** | | **Quality domain^c^** | |
| --- | --- | --- | --- | --- | --- | --- |
| Total Maternal deaths | 3 | 3 | | 2 | |  |
| Lifetime risk of maternal death | 3 | 3 | | 2 | |  |
| Maternal mortality ratio (per 100,000 live births) | 3 | 3 | | 2 | |  |
| Cause of maternal deaths (regional) | 3 | 3 | | 2 | |  |
| Stillbirth rate (per 1000 total births) | 3 | 3 | | 2 | |  |
| Neonatal deaths, as % of all <5 | 4 | 3 | | 2 | |  |
| Total Under 5 deaths | 5 | 3 | | 2 | |  |
| Under 5 mortality rate (per 1000 live births) | 5 | 3 | | 2 | |  |
| Newborn, Child, & Adolescent Causes of Death – Neonatal | 4 | 3 | | 2 | |  |
| Newborn, Child, & Adolescent Causes of Death – Under-five | 5 | 3 | | 2 | |  |
| Demand for family planning satisfied with modern methods or DSPS(MM) | 2 | 3 | | 6 | |  |
| Antenatal care (4+ visits) | 3 | 2 | | 2 | |  |
| Treatment of pregnant women living with HIV with ART | 3 | 2 | | 2 | |  |
| Neonatal tetanus protection | 4 | 2 | | 2 | |  |
| Skilled birth attendant | 3 | 2 | | 2 | |  |
| Postnatal care for mothers | 3 | 2 | | 2 | |  |
| Postnatal care for babies | 4 | 2 | | 2 | |  |
| Immunization – DTP3 | 5 | 2 | | 2 | |  |
| Immunization – Measles | 5 | 2 | | 2 | |  |
| Immunization – rotavirus | 5 | 2 | | 2 | |  |
| Diarrhoea treatment: ORS | 5 | 2 | | 2 | |  |
| Timing of first antenatal visit, women aged 15-49 | 3 | 2 | | 5 | |  |
| Intermittent preventive treatment for malaria during pregnancy 3+ | 3 | 2 | | 2 | |  |
| Institutional delivery (public, private, total) | 3 | 2 | | 2 | |  |
| Caesarean section rate (urban, rural, total) | 3 | 2 | | 2 | |  |
| Percent of children immunized with 3 doses of DTP3 | 5 | 2 | | 2 | |  |
| Percent of children immunized with 3 doses of PCV | 5 | 2 | | 2 | |  |
| Percent of children immunized against rotavirus | 5 | 2 | | 2 | |  |
| Percent of children immunized against measles | 5 | 2 | | 2 | |  |
| Full vaccination coverage (immunized for BCG, Polio3, DTP3, Measles) | 5 | 2 | | 2 | |  |
| Immunized against HPV | 3 | 2 | | 2 | |  |
| Careseeking for ARI Symptoms: Percent of children under 5 years with symptoms of acute respiratory infection taken to appropriate health provider | 5 | 2 | | 2 | |  |
| Percent of children under 5 years with diarrhoea - diarrhoea treatment: with ORS | 5 | 2 | | 2 | |  |
| Percent of children under 5 years with diarrhoea - diarrhoea treatment: with ORS + zinc | 5 | 2 | | 2 | |  |
| Careseeking for fever | 5 | 2 | | 5 | |  |
| Malaria diagnostics in children under- five | 5 | 2 | | 5 | |  |
| Number of children not vaccinated with DTP1 | 5 | 2 | | 4 | |  |
| Legal status of abortion | 2 | 1 | | 7 | |  |
| Maternity protection (Convention 183) | 3 | 1 | | 7 | |  |
| Costed national implementation plans for maternal, newborn and child health available | 1 | 1 | | 7 | |  |
| Maternal death review | 3 | 2 | | 4 | |  |
| Civil society involvement in review of national maternal, newborn and child health programmes | 1 | 1 | | 7 | |  |
| ODA+ flows to RMNCH per capita | 1 | 1 | | 7 | |  |
| ODA+ to MNH per birth | 1 | 1 | | 7 | |  |
| ODA+ to CH per child U5 | 1 | 1 | | 7 | |  |
| ODA+ to RH per woman of reproductive age | 1 | 1 | | 7 | |  |
| ODA+ to RMNCH other | 1 | 1 | | 7 | |  |
| Density of doctors, nurses, and midwives (per 10,000 population) | 1 | 1 | | 2 | |  |
| Midwives authorized for specific tasks | 1 | 1 | | 2 | |  |
| contraceptive prevalence rate | 2 | 2 | | 2 | |  |
| Vitamin A supplementation coverage | 3 | 2 | | 2 | |  |
| Perioperative mortality rate | 3 | 3 | | 2 | |  |
| Obstetric and gynecological admissions owing to abortion | 2 | 2 | | 2 | |  |
| Institutional mortality ratio | 3 | 3 | | 2 | |  |
| Prevalence of stunting among children under 5 years of age (SDG 2.2.1) | 5 | 3 | | 2 | |  |
| Current country health expenditure per capita (including specifically on RMNCAH) financed from domestic sources | 1 | 1 | | 7 | |  |
| Number of countries with laws and regulations that guarantee women aged 15-49 access to sexual and reproductive health care, information and education (SDG 5.6.2) | 1 | 1 | | 7 | |  |
| Proportion of children under 5 years of age whose births have been registered with a civil authority (SDG 16.9.1) | 5 | 2 | | 7 | |  |
| Proportion of ever-partnered women and girls aged 15 and older subjected to physical, sexual or psychological violence by a current or former intimate partner in the previous 12 months (SDG 5.2.1) and proportion of young women and men aged 18-29 who experienced sexual violence by age 18 (SDG 16.2.3) | 1 | 2 | | 7 | |  |
| Percentage of population using safely managed sanitation services including a hand-washing facility with soap and water (SDG 6.2.1) | 1 | 2 | | 4 | |  |

a: 1= General; 2 = Reproductive; 3 = Maternal; 4 = newborn; 5 = Child

b: 1= Inputs; 2 = Outputs; 3 = Outcomes

c: 1= Efficiency; 2 = Effectiveness; 3 = Equity, 4 = safety; 5 = timeliness; 6 = Patient centered; 7 = Cannot be categorized
