# Supplementary material for: Strengthening provider accountability: A scoping review of accountability/monitoring frameworks for quality of RMNCH care
Source: PLOS Glob Public Health. 2023 Nov 9;3(11):e0001968. doi: 10.1371/journal.pgph.0001968 (PMC10635430; doi:10.1371/journal.pgph.0001968)
Supplement: Attachment — Submitted filename: Zeng-reponses-to-comments.docx [file pgph.0001968.s004.docx]

Responses to reviewers’ comments

We appreciate the reviewers’ insightful comments. Below we explain, point by point, how we dealt with the comments. To make it easier for the reviewers, we have left the text of the reviewers and added our response in blue right below each of the comments.

Reviewer #1: The authors did a good work on reviewing the existing literature on monitoring frameworks for RMNCH. Methods for identifying and assessing the frameworks is clear and seems fit for purpose. The identified frameworks are well summarised and assessed clearly. Results are clear and easy to read. Conclusions are according to findings. If a country is looking into developing a monitoring framework for RMNCH services this paper does a good job in summarising and describing what is available.

1. The paper also includes some case studies that are mentiones in the introduction and methods, but not presented in the results nor in the discussion. The annex with the case studies is quite bland and does not add value to the rest of the article. Authors should seriously consider removing this part and probably use it in anothe publication.

Response: Thanks for the suggestion. As suggested, we have removed the three case studies from the appendix, and the associated description from the method and discussion.

Reviewer #2: This paper has promise, but needs to be strengthened in a few places.

1. The authors argue that Quality of Care is a ‘method’--- more needs to be added to this assertion and the evidence base that support QoC as an approach.

Response: Thanks for catching it. QoC is one of the intermediate objectives of the health system. We revised the first sentence in the abstract to “Increasing health providers’ accountability is an important element in improving quality of care (QoC) for reproductive, maternal, neonatal, and child health (RMNCH), so as to improve health outcomes of the population in many low- and middle-income countries (LMICs).”

2. The authors make an important link between QofC and accountability but the reviewer suggests using references related to accountability in the health space as opposed to what they have there. Suggest adding more language about why accountability is an important component that is underpinning QofC. There are some important pieces out there on this that should be referenced.

Response: Thanks for the suggestions. As suggested, we have changed the references in the introduction with a focus on references on accountability applied in health areas.

We also added the following sentences in the introduction to emphasize that accountability is an underpinning element of QoC: *“Accountability in healthcare is often concerned with setting up regulations to stipulate: (1) to whom health workers are held accountable; (2) which activities they are accountable for; and (3) the procedures by which their accountability is evaluated. [6] The concept of accountability has been widely used for quality of care and patient safety.[4] In fact, accountability is regarded as an important and integrated element in supporting continuous quality improvement (CQI) in healthcare,[7, 8] as one of the steps in CQI is to assess where measured outcomes meet the expectation (check) in CQI’s Plan-Do-Check-Act (PDCA) cycle. Thus, it requires the development of valid accountability measures to guide quality improvement.[9] “*

Methods related comments:

3. Authors should define global monitoring initiatives and clarify selection criteria. There needs more explanation on why/ how those specific ones were chosen.

Response: Thanks very much for the suggestion. The selection of the global monitoring initiatives was primarily informed by two studies conducted by Moller and Hilber, respectively. In Moller’s study, the selection of global RMNCH initiatives was based on the consultation with the WHO’s technical group on mother and newborn information for tracking outcomes and results (MoNITOR). We reviewed the initiatives examined by Moller et al. and assessed the appropriateness in the context of this study on measurement indicators for quality improvement. In the end, we selected four initiatives out of nine. We also further conducted a search on Google using “global initiatives”, “RMNCH”, “accountability” and “monitoring” and did not find any programs related to global monitoring efforts for RMNCH.

As suggested, we have provided more descriptions of how specific initiatives were identified and the inclusion criteria. After we described the four selected initiatives, we added the following sentences: “*For example, nine global monitoring initiatives related to RMNCH services were identified by Moller[12] while Hilber et al. leveraged the Global Strategy for Women, Children’s, Adolescents’ Health to propose potential refinement of the accountability measurement framework for global health initiatives.[11] The following criteria were applied to select specific global monitoring initiatives to review: (1) the initiative should primarily concern maternal and/or child health; (2) the initiative concerns developing or using monitoring indicators for maternal and/or child health; (3) the monitoring indicators should contain a significant number of indicators measuring the quality of care for maternal and/or child health; and (3) the initiative, along with its monitoring framework, should be applied to multiple countries and/or to the global level*.”

4. Suggest that authors explain/justify why the scoping approach was used versus systematic review, and add in the definition/criteria for using the scoping review methodology.

Response: As suggested, we provided the justification for the use of the scoping review approach. The main reference of the justification is <https://bmcmedresmethodol.biomedcentral.com/articles/10.1186/s12874-018-0611-x>.

We added the following to the choice of the scoping review: “*Because the main purpose of this study is to gauge the adequacy and complexity of monitoring indicators measuring the quality of care for maternal and child services at the global level and to identify knowledge gaps, scoping reviews were recommended and used in this study*. [10]”, and added the reference.

5. In search approach: Use of the word quick (quick review, quick search) does not provide enough information on exclusion/inclusion criteria

Response: Thanks. We removed the word of “quick” from the sentence, conducted a search again, and found no relevant articles.

We added the inclusion criteria for the selection of initiatives as explained in our response to the reviewer’s comment 3.
